# Chelsea Hospital and Women Workers

**Published:** 1912-06-29

**Authors:** Helen Ilchester, Eleanor Esher

**Affiliations:** President of Ladies' Committee.; Vice-President of Ladies' Committee.


*To the Editor of* THE HOSPITAL.

### Chelsea Hospital and Women Workers.

*To the Editor of* THE HOSPITAL.

SIR,—The letter which was recently published in the lay press on behalf of the Chelsea Hospital for Women and its Rebuilding Fund mentioned the utter inadequacy in all departments of the present accommodation. One of the instances given was that of the provision for the nursing staff, who have to sleep under three separate roofs. The plans for the new hospital have the merit of comprising a distinct building for the Nurses' Home, separated from the hospital by a small garden. It is suggested that a special fund be collected among women only for the building of this Nurses' Home. The cost will be about £8,000. The work done by the hospital for patients suffering from the many maladies to which women are peculiarly liable is so well known as hardly to need reference. What is not so well known, except to members of the Ladies' Committee and others who see a good deal of the patients, is the important share in the results achieved which must be accredited to the nursing staff. It is constantly brought to the knowledge of the Committee that the zeal and devotion with which the nurses perform their duties are accompanied by unfailing kindness and consideration for their charges.

The building of the Nurses' Home seems to offer an excellent opportunity for women to show their appreciation of this good work performed by women workers for suffering members of their own sex. Such a memorial may well serve to perpetuate the kind feeling which has fortunately long been a guiding principle to the nursing staff of the Chelsea Hospital for Women. Contributions to this Nurses' Home Rebuilding Fund are invited, and will be most gratefully received by either of the undersigned at the Hospital, Fulham Road, S.W.—Yours faithfully,

HELEN ILCHESTER,
President of Ladies' Committee.
ELEANOR ESHER,
Vice-President of Ladies' Committee.

